# Clinical Feasibility and Outcomes of Surgeon-Performed Laparoscopic-Guided Subcostal Transversus Abdominis Plane Block in Laparoscopic Cholecystectomy: Prospective Observational Study

**DOI:** 10.2196/87622

**Published:** 2026-03-25

**Authors:** Sarun Mahasupachai, Thawatchai Tullavardhana

**Affiliations:** 1 Department of Surgery, Faculty of Medicine, Srinakharinwirot University, Ransit - Nakhon nayok Road, Ongkharak, Nakhon nayok, 26120, Thailand, 66 879791989

**Keywords:** laparoscopic cholecystectomy, minimally invasive surgery, postoperative pain, transversus abdominis plane block, enhanced recovery after surgery

## Abstract

**Background:**

Laparoscopic-guided subcostal transversus abdominis plane (TAP) block has been introduced as a surgeon-performed approach to postoperative analgesia in laparoscopic cholecystectomy (LC), allowing direct visual confirmation of local anesthetic delivery without ultrasound guidance. However, evidence regarding its clinical outcomes, particularly in patients with complicated gallstone disease, remains limited.

**Objective:**

This study aimed to evaluate postoperative analgesic outcomes and identify factors associated with opioid requirement following laparoscopic-guided subcostal TAP block.

**Methods:**

A prospective observational study was conducted between November 2023 and October 2024 at Srinakharinwirot University Hospital, Thailand. Patients (aged 18‐80 years) undergoing LC for uncomplicated or complicated gallstone disease received a laparoscopic-guided subcostal TAP block with 0.25% bupivacaine. Postoperative pain was assessed using the Visual Analog Scale at 2, 4, 6, 8, 12, and 24 hours. Morphine administration within the first 24 hours was recorded. Associations between perioperative variables and opioid requirement were analyzed using univariate and exploratory multivariable logistic regression.

**Results:**

A total of 42 patients were included in the analysis. Of these, 21 (50%) did not require postoperative opioids, while the remaining patients (n=21, 50%) received a mean cumulative morphine dose of 3.86 (SD 1.39) mg within 24 hours. Pain scores were lower during the early postoperative period (2, 4, and 12 h) in patients who did not require opioids. Higher American Society of Anesthesiologists classification was independently associated with postoperative morphine requirement (odds ratio 6.51, 95% CI 1.37‐30.96; *P*=.01). No major complications or local anesthetic toxicity were observed.

**Conclusions:**

In this prospective observational cohort, laparoscopic-guided subcostal TAP block may be associated with favorable early postoperative analgesic profiles and relatively low opioid requirements after LC, including in patients with gallstone-related complications. Higher American Society of Anesthesiologists classification may be associated with increased opioid demand, highlighting the importance of individualized, risk-adapted analgesic strategies. Although limited by the absence of a control group and modest sample size, these findings support the clinical feasibility of surgeon-performed TAP block for consideration within multimodal analgesia approaches in enhanced recovery after surgery–oriented perioperative care.

## Introduction

Laparoscopic cholecystectomy (LC) is the standard surgical approach for gallstone disease, offering distinct advantages over open cholecystectomy, including shorter recovery times and earlier return to normal activities [1]. Despite these benefits, LC is associated with moderate to severe postoperative pain, particularly within the first 24 hours, often necessitating opioid analgesia [2]. High-dose opioid use, however, is frequently complicated by nausea, vomiting, dizziness, abdominal distension, and urinary retention, which may delay recovery and prolong hospitalization [3,4].

Enhanced recovery after surgery (ERAS) protocols have been widely implemented to optimize perioperative care and expedite recovery. Multimodal analgesia represents a cornerstone of these protocols, aiming to minimize opioid use while maintaining effective pain control [5,6]. Within this framework, the subcostal transversus abdominis plane (TAP) block has emerged as a valuable component of multimodal analgesia, providing targeted pain relief following LC. When performed under ultrasound guidance using 0.25% bupivacaine, this block reliably anesthetizes thoracic (T7-T12) and lumbar (L1) nerves, thereby improving pain control and facilitating earlier mobilization [7,8]. Nevertheless, its dependence on anesthesiologist expertise and specialized equipment limits feasibility in certain clinical environments.

To address these limitations, the laparoscopic-guided subcostal TAP block has been developed as a surgeon-performed technique seamlessly incorporated into the operative workflow. Under direct laparoscopic visualization, local anesthetic can be precisely delivered into the TAP, providing consistent parietal analgesia while obviating the need for ultrasound equipment or additional personnel. This method has been demonstrated to be safe, efficient, and time-effective, offering a practical alternative for postoperative pain control following LC [9].

Nevertheless, most previous studies evaluating TAP block for LC have primarily focused on patients with uncomplicated gallstone disease and were conducted in controlled trial settings. A recent systematic review and meta-analysis demonstrated that TAP block is effective in reducing postoperative pain and opioid consumption after LC, with most included studies using ultrasound-guided techniques [10]. In contrast, this study evaluates a surgeon-performed, laparoscopic-guided subcostal TAP block integrated into routine operative workflow.

This study aimed to assess the clinical feasibility and outcomes of surgeon-performed, laparoscopic-guided subcostal TAP block for postoperative pain management in patients undergoing LC for both uncomplicated and complicated gallstone disease, including acute cholecystitis and biliary tract obstruction. Additionally, perioperative predictors of postoperative opioid requirement were explored.

## Methods

### Overview

A single-center observational study was conducted at the Department of Surgery, Faculty of Medicine, Srinakharinwirot University, Thailand, between November 2023 and October 2024. Eligible patients were aged 18 to 80 years and diagnosed with symptomatic cholelithiasis or gallstone-related complications. Uncomplicated gallstone disease was defined as symptomatic cholelithiasis or chronic cholecystitis without evidence of systemic inflammation or biliary complications. Complicated gallstone disease was defined as gallstone-related conditions associated with acute inflammation or biliary obstruction, including acute cholecystitis, acute cholangitis, or biliary obstruction requiring endoscopic retrograde cholangiopancreatography. Patients who were converted to open surgery or had a known allergy to bupivacaine were excluded. The target sample size was 40 patients; however, to enhance reliability, a total of 50 patients were enrolled.

### Study Design Declaration

The initial ethics-approved protocol was designed as a randomized comparison between subcostal TAP block and port-site local infiltration. However, due to limited patient recruitment, randomization could not be executed. This report therefore represents an observational analysis of patients who received the surgeon-performed, laparoscopic-guided subcostal TAP block in accordance with the originally approved protocol. No additional procedures, interventions, or deviations from the ethics approval were undertaken.

### Ethical Considerations

This study was approved by the Institutional Ethics Committee of Srinakharinwirot University (ethics code: SWUEC-004/2566F). Written informed consent was obtained from all participants prior to enrollment, and all patient data were collected and managed in accordance with institutional and international standards for data confidentiality and ethical research practice.

The study was retrospectively registered with the Thai Clinical Trials Registry (TCTR20250314002) following a change in study execution from the originally approved randomized protocol to a prospective observational design. Importantly, no protocol deviations occurred beyond the scope approved by the institutional ethics committee.

### Laparoscopic-Guided Subcostal TAP Block Technique

LC was performed under general anesthesia. Intraoperative analgesia was standardized according to the institutional protocol and was not analyzed separately. Prior to skin incision, 5 mL of 0.25% bupivacaine was infiltrated at the umbilical site for local analgesia. A 12-mm umbilical port was inserted for the laparoscopic camera, and pneumoperitoneum was established at an intra-abdominal pressure of 8 to 12 mmHg.

After establishment of pneumoperitoneum, the right upper quadrant was inspected under laparoscopic visualization. The injection point was identified at the right subcostal region, approximately 2 to 3 cm inferior to the costal margin and lateral to the midline, corresponding to the junction between the posterior rectus sheath and the transversus abdominis muscle.

**Figure 1.**
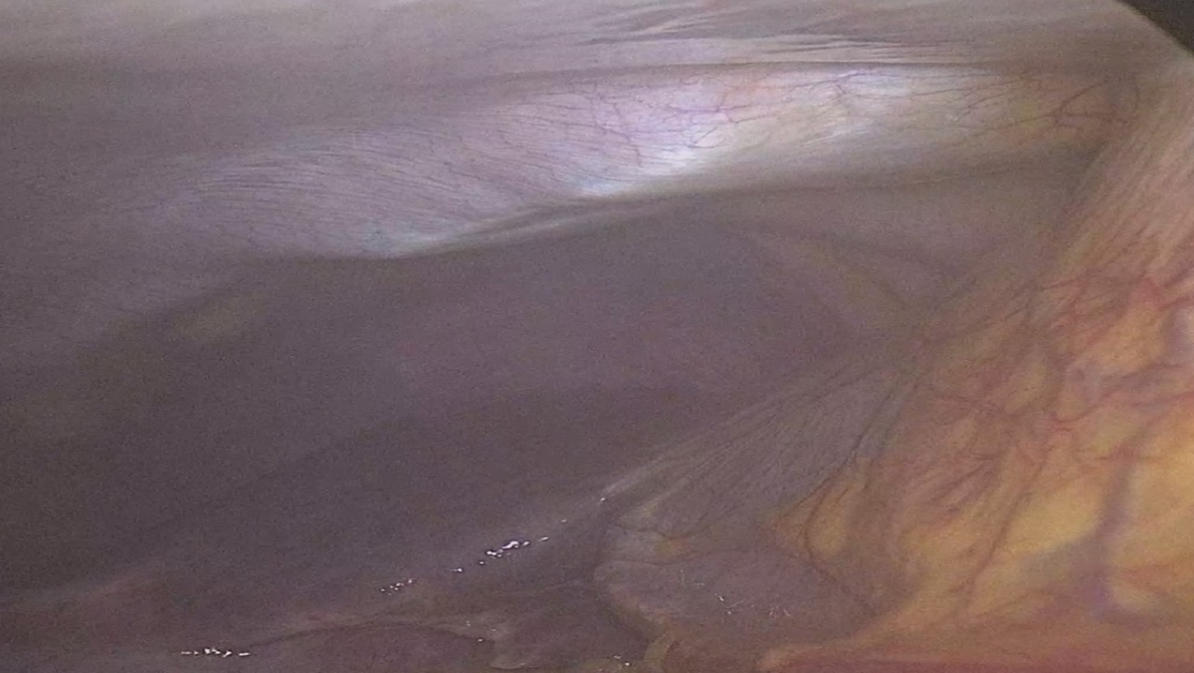
Illustration of Doyle bulge observed during laparoscopic-guided subcostal transversus abdominis plane block.

Under direct laparoscopic visualization, a long spinal needle was inserted percutaneously toward the TAP, with initial advancement at an angle of approximately 60 to 80 degrees relative to the abdominal wall. Upon reaching the target fascial plane, the needle angle was adjusted to approximately 30 degrees to facilitate controlled anesthetic delivery. After negative aspiration, 20 mL of 0.25% bupivacaine was injected incrementally. Correct local anesthetic deposition was inferred from laparoscopic visualization of the Doyle bulge, indicating separation of the posterior rectus sheath and transversus abdominis muscle as a surrogate marker of appropriate fascial plane injection (Figure 1).

In our contemporary LC practice, routine use of a 12-mm epigastric working port has been replaced by three 5-mm working ports positioned along the right subcostal margin, in addition to the 12-mm umbilical optical port. This port configuration reflects an evolving minimally invasive approach aimed at reducing port-site trauma while maintaining adequate operative ergonomics and visualization. After cholecystectomy, the gallbladder was retrieved in a sterile bag through the 12-mm umbilical port. A supplementary video demonstrating the laparoscopic-guided subcostal TAP block technique is provided in Multimedia Appendix 1.

### Postoperative Pain Management and Monitoring

All patients received standardized postoperative analgesia. Oral acetaminophen (500 mg every 6 hours) and naproxen (250 mg twice daily) were prescribed as first-line agents; tramadol (50 mg twice daily) was substituted for patients with nonsteroidal anti-inflammatory drug intolerance. Persistent pain with a Visual Analog Scale (VAS) score >5 despite oral medication was managed with intravenous morphine (0.1 mg/kg every 4 hours as needed). Ondansetron (4 mg every 8 hours as required) was given for nausea and vomiting prophylaxis.

Pain intensity was evaluated using the VAS (0=no pain and 10=worst pain) at 2, 4, 6, 8, 12, and 24 hours postoperatively and during mobilization. Total morphine consumption and opioid-related adverse events (nausea, vomiting, and urinary retention) were recorded within the first 24 hours. Patients were observed for possible bupivacaine toxicity during this period.

### Clinical Variables

Data collection included patient demographics, comorbidities, laboratory parameters, operative details, postoperative outcomes, and histopathological findings. Laboratory parameters analyzed in this study were preoperative values obtained within 24 hours before surgery. Operative variables comprised surgical duration, estimated blood loss, and intraoperative complications.

Postoperative variables included pain scores, total morphine consumption, and postoperative complications. All data were prospectively recorded in a predesigned spreadsheet (Microsoft Excel) for statistical analysis. The dataset was stored in password-protected files on secure institutional computers, with access restricted to study investigators only. Patient identifiers were removed and replaced with coded study numbers to ensure confidentiality.

### Statistical Analysis

Statistical analysis was conducted using SPSS Statistics (version 27.0; IBM Corp). Descriptive statistics (mean, SD, frequencies, and percentages) were used to summarize demographic and clinical data. Comparisons between subgroups were conducted using the chi-square or Fisher exact test for categorical variables and the independent 2-tailed *t* test (or 1-way ANOVA where applicable) for continuous variables.

Exploratory multivariable logistic regression was applied to assess potential associations between perioperative factors and postoperative morphine requirement, acknowledging the limited sample size. Variables included in the multivariable model were selected based on clinical relevance and prior literature, rather than solely on statistical significance in univariate analyses. Given the limited number of outcome events, the analysis was conducted with a restricted number of covariates to maintain an acceptable events per variable ratio and minimize the risk of overfitting. A 2-tailed *P* value <.05 was considered statistically significant.

## Results

### Overview

During the study period, 50 patients underwent LC. In total, 8 (16%) patients were excluded due to conversion to open surgery, leaving 42 (84%) patients for analysis. Of these, 21 (50%) patients required postoperative morphine within the first 24 hours. Among patients who received opioids (n=21, 50%), the mean cumulative morphine dose was 3.86 (SD 1.39) mg.

### Patient Characteristics

Baseline demographic and clinical characteristics are summarized in Table 1. There were no significant differences between the morphine-required and morphine-free groups in age (*P*=.55), BMI (*P*=.55), or sex distribution (*P*=.22). Comorbid conditions were more frequent in the morphine-required group; however, this difference did not reach statistical significance (*P*=.10). The mean American Society of Anesthesiologists (ASA) classification was significantly higher among patients who required morphine (mean 2.14, SD 0.57 vs mean 1.67, SD 0.73; *P*=.024), reflecting a higher baseline perioperative risk profile.

**Table 1.** Baseline demographic and clinical characteristics of the study population.

Variable	Morphine required (n=21)	Morphine free (n=21)	*P* value
Age (years), mean (SD)	57.1 (14.1)	54.3 (15.8)	.55
Sex (female), n (%)	13 (61.9)	8 (38.1)	.22
BMI (kg/m²), mean (SD)	25.8 (5.3)	24.8 (4.9)	.55
ASA^a^ classification, mean (SD)	2.14 (0.57)	1.67 (0.73)	.02
Any comorbidity, n (%)	17 (81.0)	10 (47.6)	.10
Diabetes mellitus, n (%)	10 (47.6)	6 (28.6)	.21
Hypertension, n (%)	13 (61.9)	10 (47.6)	.36
Cardiovascular disease, n (%)	6 (28.6)	4 (19.0)	.48
Indication for surgery, n (%)^b^			
Symptomatic gallstone	17 (81.0)	15 (71.4)	*.*48
Acute cholecystitis	2 (9.5)	4 (19.0)	.39
Interval LC^c^ after conservative treatment of acute cholecystitis	4 (19.0)	4 (19.0)	*>*.99
Previous ERCP^d^, n (%)	6 (28.6)	5 (23.8)	*.*73

a ASA: American Society of Anesthesiologists.

b Categories are not mutually exclusive.

c LC: laparoscopic cholecystectomy.

d ERCP: endoscopic retrograde cholangiopancreatography.

### Preoperative Laboratory Parameters

Preoperative laboratory findings are presented in Table 2. All laboratory values represent measurements obtained within 24 hours prior to surgery. Patients in the morphine-required group had significantly lower baseline hemoglobin (*P*=.01) and hematocrit levels (*P*=.002). Additionally, no significant between-group differences were observed in white blood cell count, neutrophil to lymphocyte ratio, liver function tests, or serum albumin levels.

**Table 2.** Preoperative laboratory parameters.

Variable	Morphine required (n=21), mean (SD)	Morphine free (n=21), mean (SD)	*P* value
Hemoglobin (g/dL)	11.95 (1.78)	13.34 (1.60)	.01
Hematocrit (%)	35.9 (4.8)	40.2 (4.0)	*.*002
White blood cell count (×10³/µL)	11.6 (19.1)	10.7 (11.1)	*.*85
Neutrophil to lymphocyte ratio	6.92 (13.8)	4.54 (6.54)	*.*48
Aspartate aminotransferase (U/L)	41.2 (54.4)	45.2 (55.5)	*.*81
Alanine aminotransferase (U/L)	38.8 (63.7)	46.0 (69.6)	*.*73
Alkaline phosphatase (U/L)	83.8 (41.2)	95.1 (84.9)	*.*58
Total bilirubin (mg/dL)	0.70 (0.51)	0.99 (1.30)	*.*35
Serum albumin (g/dL)	4.33 (0.39)	4.21 (0.34)	*.*56

### Operative Details and Postoperative Outcomes

Operative and postoperative outcomes are summarized in Table 3. Mean operative time (mean 63.5, SD 15.6 minutes vs mean 58.4, SD 18.0 minutes; *P*=.32), estimated blood loss (mean 15.7, SD 11.3 mL vs mean 13.3, SD 12.5 mL; *P=.*52), and length of hospital stay (mean 2.57, SD 0.98 days vs mean 2.33, SD 0.86 days; *P*=.40) did not differ significantly between groups. Furthermore, no significant differences were observed between groups regarding trocar placement, operative technique, operative time, or intraoperative complications, and background analgesic regimens were comparable. Although patients with gallstone-related complications were included as indications for surgery, final histopathological examination of gallbladder specimens was reported as acute or chronic cholecystitis.

**Table 3.** Operative details, analgesic regimen, and postoperative outcomes.

Variable	Morphine required (n=21)	Morphine free (n=21)	*P* value
Analgesic regimen			
Acetaminophen+naproxen, n (%)	7 (33.3)	12 (57.1)	.12
Acetaminophen+tramadol, n (%)	14 (66.7)	9 (42.9)	.22
Operative details			
Complete cholecystectomy, n (%)	20 (95.2)	19 (90.5)	.56
Partial cholecystectomy, n (%)	1 (4.7)	2 (9.5)	.56
Operative time (minutes), mean (SD)	63.5 (15.6)	58.4 (18.0)	.32
Estimated blood loss (mL), mean (SD)	15.7 (11.3)	13.3 (12.5)	.52
Postoperative outcomes			
Postoperative complication, n (%)	1 (4.7)	1 (4.7)	.44
Nausea and vomiting, n (%)	1 (4.7)	1 (4.7)	.44
Hospital stay (days), mean (SD)	2.57 (0.98)	2.33 (0.86)	.40
Pathology: chronic cholecystitis, n (%)	20 (95.3)	17 (81.0)	.16
Pathology: acute cholecystitis, n (%)	1 (4.7)	4 (19.0)	.30

### Factors Associated With Postoperative Morphine Requirement

Factors associated with postoperative morphine requirement in exploratory multivariable analysis are summarized in Table 4. Higher ASA class was associated with increased odds of morphine use within 24 hours after surgery (*P*=*.*01; odds ratio 6.51, 95% CI 1.37‐30.96). Lower hemoglobin level demonstrated a trend toward association with morphine requirement but did not reach statistical significance (*P*=.07; odds ratio 0.58, 95% CI 0.32‐1.06). Other variables, including age, sex, gallstone-related complications, and history of endoscopic retrograde cholangiopancreatography, were not significantly associated with opioid use.

**Table 4.** Multivariable logistic regression analysis of factors associated with postoperative morphine requirement.

Variable	Odds ratio (95% CI)	*P* value
Female	1.81 (0.26-12.80)	.55
ASA^a^ classification	6.51 (1.37-30.96)	.01
Hemoglobin (per g/dL)	0.58 (0.32-1.06)	.07
Age (years)	0.98 (0.92-1.04)	.46
Gallstone-related complication	0.91 (0.16-5.15)	.91
Previous ERCP^b^	0.75 (0.12-4.56)	.75

a ASA: American Society of Anesthesiologists.

b ERCP: endoscopic retrograde cholangiopancreatography.

Given the limited number of outcome events, regression analyses were conducted within an exploratory framework constrained by events per variable considerations.

### Postoperative Pain Scores

Postoperative pain scores are summarized in Table 5. Patients who required morphine reported higher VAS scores during the early postoperative period, particularly at 2 hours (mean 3.29, SD 1.45 vs mean 1.93, SD 0.96; *P*=.009), 4 hours (mean 3.95, SD 1.39 vs mean 2.07, SD 1.00; *P*<.001), and 12 hours (mean 3.57, SD 1.44 vs mean 2.43, SD 1.06; *P*=.02). At 6 hours, 24 hours, and during mobilization, pain scores remained numerically higher in the morphine-required group but did not reach statistical significance.

**Table 5.** Comparison of postoperative pain scores between morphine-required and morphine-free groups.

Time point	Morphine required, mean (SD)	Morphine free, mean (SD)	*P* value
2 hours	3.29 (1.45)	1.93 (0.96)	.009
4 hours	3.95 (1.39)	2.07 (1.00)	<.001
6 hours	3.33 (1.43)	2.73 (1.42)	.26
12 hours	3.57 (1.44)	2.43 (1.06)	.02
24 hours	2.57 (1.21)	1.97 (0.81)	.07
During mobilization	4.29 (1.33)	3.97 (1.25)	.40

These findings descriptively reflect differences in pain experience between groups and are presented to contextualize postoperative opioid requirement rather than to infer comparative analgesic effectiveness.

## Discussion

### Principal Findings

This prospective observational study suggests a clinically relevant opioid-sparing association of laparoscopic-guided subcostal TAP block in patients undergoing LC. Approximately half of the patients (n=21, 50%) did not require postoperative opioids, while those who did (n=21, 50%) received only a modest cumulative dose (mean 3.86, SD 1.39 mg).

The observed analgesic pattern was most evident during the early postoperative period, particularly between 2 and 12 hours, which is consistent with the expected pharmacodynamic profile of 0.25% bupivacaine. Within the context of this observational cohort, these findings support the feasibility of surgeon-performed TAP block as a practical adjunct to multimodal analgesia strategies, with the potential to limit postoperative opioid exposure while maintaining adequate pain control [11-14].

The results of this study are directionally consistent with previous reports suggesting that thoracoabdominal and subcostal TAP blocks are associated with improved early postoperative pain control following LC [15-17]. Importantly, this study extends the existing literature by including patients with complicated gallstone disease, a population that has been relatively underrepresented in prior research.

From a mechanistic perspective, the observed analgesic association may be attributable to localized somatic blockade of the upper abdominal wall corresponding to trocar insertion sites. Such coverage is thought to attenuate incisional and parietal peritoneal pain, which may explain the more pronounced pain relief observed during the first 12 postoperative hours [10,18]. Clinically, these observations underscore the importance of integrating regional analgesic techniques with scheduled nonopioid coanalgesics and appropriately timed rescue analgesia to maintain adequate pain control within ERAS-oriented perioperative pathways [5,6,13,14].

Exploratory predictor analysis suggested that patient-related factors were more strongly associated with postoperative opioid requirement than intraoperative variables. Higher ASA classification was independently associated with postoperative morphine use, indicating that greater comorbidity burden may be linked to increased analgesic needs despite regional blockade. These findings support a risk-adapted approach to perioperative pain management for patients at higher risk.

Although the TAP block primarily targets somatic abdominal wall pain, unmeasured factors such as visceral pain burden, neuropathic pain components, and subtle variations in block accuracy may have influenced postoperative pain perception and opioid requirement. These factors were not objectively assessed in this study and may contribute to residual variability beyond patient-level characteristics such as ASA classification.

Beyond its analgesic association, surgeon-performed subcostal TAP block offers several practical advantages, particularly in settings with limited anesthesiology support or restricted access to ultrasound equipment. Incorporation of this technique into the laparoscopic workflow allows a consistent and equipment-independent approach to regional analgesia, aligning with broader initiatives in opioid stewardship and sustainable perioperative care [9,15-17,19].

Several limitations should be acknowledged. The single-center, nonrandomized design limits causal inference, and the absence of a control group precludes direct comparison with standard port-site local anesthetic infiltration. The modest sample size restricts statistical precision; therefore, multivariable analyses were conducted within an exploratory framework with selective variable inclusion to reduce the risk of overfitting.

In addition, no formal postoperative sensory testing was performed to objectively verify block success, primarily due to practical considerations within the perioperative workflow and the study’s focus on clinically relevant outcomes. Instead, correct local anesthetic deposition was inferred from laparoscopic visualization of the Doyle bulge, indicating separation of the posterior rectus sheath and transversus abdominis muscle as a surrogate marker of appropriate fascial plane injection [20,21]. However, this visual confirmation cannot fully substitute for objective sensory testing and does not confirm the extent or consistency of dermatomal blockade.

Substitution of tramadol for nonsteroidal anti-inflammatory drugs in a small number of patients may have introduced minor confounding, although perioperative analgesic protocols were otherwise standardized. In addition, psychosocial factors known to influence postoperative pain, including anxiety, pain catastrophizing, and prior opioid exposure, were not assessed [22,23]. These limitations should be considered when interpreting the findings.

Despite these constraints, this study has several notable strengths. The prospective data collection, use of standardized perioperative analgesic pathways, and inclusion of patients with both uncomplicated and complicated gallstone disease enhance the clinical relevance of the study. Overall, the findings indicate that surgeon-performed, laparoscopic-guided subcostal TAP block is a technically straightforward and reproducible adjunct within multimodal analgesia strategies. The observed early opioid-sparing association supports the feasibility of considering this technique in ERAS-oriented perioperative pathways and broader opioid reduction efforts.

The relatively longer hospital stay observed in this cohort reflects local institutional practice, in which LC is not routinely performed as a day-case procedure. Inclusion of patients with complicated gallstone disease required preoperative admission and postoperative observation for safety. In addition, routine preoperative admission at least 1 day prior to surgery, according to institutional protocol, contributed to the overall length of hospital stay.

Future research should include multicenter randomized controlled trials comparing laparoscopic-guided TAP block with standard port-site local anesthetic infiltration. On the basis of the observed effect estimates, a sample size of approximately 80 to 100 patients per arm may provide 80% power to detect a 1-point difference in mean VAS pain score at an α level of .05. Incorporation of cost-effectiveness analyses, patient-reported outcome measures, and longer-term follow-up would further clarify the clinical value, scalability, and role of this technique in minimally invasive surgery.

### Conclusions

This study suggests that laparoscopic-guided subcostal TAP block may be associated with lower early postoperative pain scores and reduced opioid requirements in patients undergoing LC, including those with gallstone-related complications. Although limited by sample size, the findings support the feasibility of considering surgeon-performed subcostal TAP block as part of multimodal analgesia strategies within ERAS-oriented perioperative pathways.

## Supplementary material

10.2196/87622 Multimedia Appendix 1 Laparoscopic-guided subcostal transversus abdominis plane (TAP block).

## Abbreviations

ERAS: enhanced recovery after surgery
LC: laparoscopic cholecystectomy
TAP: transversus abdominis plane
VAS: Visual Analog Scale
